# Chronic social defeat stress impairs goal-directed behavior through dysregulation of ventral hippocampal activity in male mice

**DOI:** 10.1038/s41386-021-00990-y

**Published:** 2021-03-10

**Authors:** Keitaro Yoshida, Michael R. Drew, Anna Kono, Masaru Mimura, Norio Takata, Kenji F. Tanaka

**Affiliations:** 1 Department of Neuropsychiatry, Keio University School of Medicine, Tokyo, Japan; 2 Center for Learning and Memory, Department of Neuroscience, The University of Texas at Austin, Austin, TX USA

**Keywords:** Motivation, Depression

## Abstract

Chronic stress is a risk factor for a variety of psychiatric disorders, including depression. Although impairments to motivated behavior are a major symptom of clinical depression, little is known about the circuit mechanisms through which stress impairs motivation. Furthermore, research in animal models for depression has focused on impairments to hedonic aspects of motivation, whereas patient studies suggest that impairments to appetitive, goal-directed motivation contribute significantly to motivational impairments in depression. Here, we characterized goal-directed motivation in repeated social defeat stress (R-SDS), a well-established mouse model for depression in male mice. R-SDS impaired the ability to sustain and complete goal-directed behavior in a food-seeking operant lever-press task. Furthermore, stress-exposed mice segregated into susceptible and resilient subpopulations. Interestingly, susceptibility to stress-induced motivational impairments was unrelated to stress-induced social withdrawal, another prominent effect of R-SDS in mouse models. Based on evidence that ventral hippocampus (vHP) modulates sustainment of goal-directed behavior, we monitored vHP activity during the task using fiber photometry. Successful task completion was associated with suppression of ventral hippocampal neural activity. This suppression was diminished after R-SDS in stress-susceptible but not stress-resilient mice. The serotonin selective reuptake inhibitor (SSRI) escitalopram and ketamine both normalized vHP activity during the task and restored motivated behavior. Furthermore, optogenetic vHP inhibition was sufficient to restore motivated behavior after stress. These results identify vHP hyperactivity as a circuit mechanism of stress-induced impairments to goal-directed behavior and a putative biomarker that is sensitive to antidepressant treatments and that differentiates susceptible and resilient individuals.

## Introduction

Impaired motivation is a cardinal symptom of depression [1, 2]. Among depression symptoms, diminished motivational drive is one of the strongest predictors of a depression diagnosis [3]. Motivational impairments potentially involve perturbations to at least two distinct motivational processes: changes in the hedonic impact (liking or pleasure) derived from rewards (RWs), and changes in cost-benefit calculations that control the willingness to engage in and sustain effortful behaviors required to earn a RW [4, 5]. It can be difficult to disentangle these processes experimentally [6]. Although there is indirect evidence suggesting that hedonic responses to RW are blunted in depressed patients, studies directly assessing pleasure responses have not yielded evidence of impaired hedonic impact in depressed patients [7]. In contrast, depressed patients do exhibit deficits in effortful RW-based tasks [8], leading some researchers to conclude that motivational deficits in depressed patients reflect a decreased willingness to expend effort to obtain RWs [9].

Despite this evidence from patient studies, research in animal models for depression has tended to focus on hedonic RW. In two well-validated rodent models for depression, chronic social defeat stress (SDS) [10] and chronic unpredictable stress [11], stress-exposed animals display a reduced preference for sweet RWs, which has been interpreted as reflecting blunted hedonic impact. Studies linking stress and motivation have typically focused on striatal regions implicated in hedonic RW processing [12]. There is also evidence that chronic stress can impair goal-directed motivation in operant tasks that require sustained responding to earn RW [13, 14]. However, the mechanisms of stress effects on goal-directed motivation are unknown.

Recent evidence, however, identifies vHP, a region believed to be highly sensitive to both stress and antidepressant medications [15–19], as a critical regulator of goal-directed motivation. In tasks that require sustained effort to earn a RW, ventral CA1 (vCA1) population activity is suppressed during periods of sustained effort, and optogenetic manipulations demonstrate that this suppression is required for successful completion of the task [20]. Based on prior evidence linking chronic stress to hippocampal hyperactivity [21, 22], we hypothesized that chronic stress exposure might impair goal-directed motivation by interfering with the suppression of vCA1 activity.

Although stress is a major risk factor for mood disorders [23], there is considerable inter-individual variation in the susceptibility versus resilience to stress [24]. This heterogeneity has been modeled in animals using the chronic SDS paradigm [10]. In mice, exposure to chronic SDS induces social withdrawal, but among stress-exposed animals there are susceptible and resilient populations. In recent years considerable effort has been directed at identifying neural and molecular mechanisms of stress susceptibility and resilience [25, 26]. It remains unclear, however, whether stress effects on motivation are equally heterogeneous and whether the mechanisms controlling motivational resilience and susceptibility are the same as those mediating stress effects on social behavior.

Here, we demonstrate that social defeat stress (R-SDS) impairs goal-directed motivation in male mice. However, the population of stress-exposed animals can be stratified into susceptible and resilient populations. Using fiber photometric recordings of bulk neural activity, we show that stress effects on goal-directed motivation are associated with perturbations in ventral hippocampal activity, and stress-induced motivational impairments can be alleviated with appropriately timed manipulation of vHP activity. Finally, we demonstrate that two distinct classes of antidepressants, a slow-acting serotonin selective reuptake inhibitor (SSRI) and rapid/prolonged-acting ketamine, rescue stress-induced impairments in motivated behavior and hippocampal function.

## Methods and materials

### Animals

All animal procedures were conducted in accordance with the National Institutes of Health *Guide for the care and use of laboratory animals* and approved by the Animal Research Committee of Keio University. Experiments were carried out using 8- to 14-week-old male mice. All mice were maintained with 12:12-h light/dark cycle (lights on at 8 am) and the behavioral experiments were conducted during the light phase. Htr5B-YC mice (*Htr5B*-tTA::tetO-YCnano50 double transgenic mice) were obtained by crossing *Htr5B*-tTA mice [27] and tetO-YCnano50 mice [28]. Htr5B-ArchT mice (*Htr5B*-tTA::tetO-ArchT-EGFP double transgenic mice) were obtained by crossing *Htr5B*-tTA mice and tetO-ArchT-EGFP mice [29]. The genetic background of all transgenic mice was mixed C57BL6 and 129SvEvTac. Genotyping for *Htr5B*-tTA, tetO-YCnano50, and tetO-ArchT was previously described [27–29]. Male ICR mice over 13 weeks of age were purchased from Oriental Yeast Co, Ltd, Japan.

### Surgical procedure

Surgeries were performed using a stereotaxic system (SM-6M-HT, Narishige). Mice were anesthetized with a mixture of ketamine and xylazine (100 mg/kg and 10 mg/kg, respectively, intraperitoneal [i.p.]). Their body temperature was maintained at 37 ± 0.5 °C using a heating pad (FHC-MO, Muromachi Kikai) during surgery. For optical recordings at the vCA1, Htr5B-YC mice were unilaterally (right side or left side) implanted with an optical fiber cannula (CFMC14L05, 400 μm, 0.39 NA; Thorlabs) into the vCA1 (−3.08 mm anteroposterior (AP), 3.5 mm mediolateral (ML) from bregma, 3.7 mm dorsoventral (DV) from the brain surface) according to the atlas of Paxinos and Franklin [30]. For optogenetic manipulations, Htr5B-ArchT mice were bilaterally implanted with 200 μm core diameter optical fiber (0.39 NA, Thorlabs) into the vCA1 (−3.08 mm AP, ±3.5 mm (ML), 3.7 mm DV).

### Fixed ratio (FR) operant task

The method for the food-seeking lever-press task has been described previously [20]. Mice were housed individually under conditions of food restriction. Their body weights were maintained at 85% of initial body weight. Behavioral trainings and tests were performed in an aluminum operant chamber measuring W22 × D26 × H18 cm (Med Associates) under constant darkness. The apparatus was controlled by a computer program written in the MED-PC language (Med Associates). A food dispenser flanked by two retractable levers was located on the floor. The lever on the left side is designated as “active” (triggering delivery of a food RW), and the one on the right is “inactive” (no relation to food RW). Each trial began with the presentation of two levers (trial start [TS]). After mice pressed the active lever (lever press [LP]), the levers were retracted and one food pellet (20 mg each, dustless precision pellets, Bio-serv) was delivered as a RW. After the food delivery, 30 s of intertrial interval (ITI) was added, during which levers were retracted, followed by the automatic starting of the next trial. The ITI allows time for mice to consume the food pellet. The training started with a FR-1 schedule, in which the mice obtained one food RW after each active LP. Once the animals were able to obtain 50 RWs within 60 min, the training progressed to an FR-2 schedule, in which two active LPs were needed for a couple of sessions. The training moved on to an FR-3 schedule when the animals could obtain 50 trial RWs in 60 min. Each FR training session lasted 1 h or when 100 pellets had been delivered. In an FR-5 or FR-10, the lever presentation was lasted for 30 s. If the mice pressed performed the required number of active LPs during the lever presentation, the levers were retracted and one food pellet was delivered (“successful trial”). After the food delivery, there was a 30-s ITI, during which levers were retracted, followed by the automatic starting of the next trial. If the mouse pressed the active lever at least once, but did not make the required number of presses during lever presentation, the levers were retracted and trial ended (“incomplete trial”). Incomplete trials were not included in the analysis of completion latency. If the mouse never pressed the active lever during lever presentation, the levers were retracted and the trial ended (“omission trial”). On average, it took 20 days for surgery, recovery, and the entire behavioral procedure including training.

### Fiber photometry

The method for fiber photometry has been described previously [31]. An exciting light (435 nm; silver-LED, Prizmatix) was reflected off a dichroic mirror (DM455CFP; Olympus), focused with a 20 × objective lens (NA 0.39, Olympus), and coupled into an optical fiber (M79L01, Φ 400 μm, 0.39 NA; Thorlabs) through a pinhole (Φ 400 μm). LED power was <100 μW at the fiber tip. Emitted cyan and yellow fluorescence from YCnano 50 was collected via an optical fiber cannula, divided by a dichroic mirror (DM515YFP; Olympus) into cyan (483/32 nm band-path filters, Semrock) and yellow (542/27 nm), and detected by each photomultiplier tube (H10722-210, Hamamatsu Photonics). The fluorescence signals in addition to TTL signals from behavioral settings were digitized by a data acquisition module (cDAQ-9178, National Instruments), and simultaneously recorded using a custom-made LabVIEW program (National Instruments). Signals were collected at a sampling frequency of 1000 Hz.

### Optogenetic manipulation during operant task

The optogenetic methods have been described previously [20]. Two sessions were conducted with stimulation (yellow) or control (blue), counterbalanced. Optical fibers (NA 0.39, Thorlabs) were inserted bilaterally through the guide cannulae. Yellow (575 nm) and blue (475 nm) light were generated by a SPECTRA 2-LCR-XA light engine (Lumencor). The yellow and blue light power intensities at the tip of the optical fiber were 3–4 mW and 2–3 mW, respectively. The light was controlled by the TTL pulses generated by MED-PC (Med Associates). In FR task, one session, consisted of 50 trials (stimulation; 25 trials, control; 25 trials, counterbalanced), was conducted.

### Repeated social defeat stress (R-SDS)

Social defeat (SD) mice were exposed to chronic SDS as previously described [32]. We used only male mice because aggressor mice do not attack female mice if there is no genetic manipulation introduced in the aggressor mice. At least 1 week before beginning the social defeat experiment, all resident ICR mice more than 13 weeks of age were singly housed on home cage (18.2 cm × 26.0 cm × 12.8 cm). Prior to R-SDS, male ICR mice were screened for their aggressiveness to a novel C57BL/6J mouse for 3 min daily for 3 days. We evaluated the aggression of the ICR mice by the latency and the number of attacks during the observation period, and only used those showing stable aggression for further experiments. After evaluating behavioral parameters and vCA1 activity of test mice, we conducted R-SDS. These mice were then transferred to the home cage of a male ICR mouse for 20 min daily for 10 days. The pairs of defeated and aggressor mice were randomized daily to minimize the variability in the aggressiveness of aggressor mice. After the 20 min defeat episode, the mice were returned to their home cages and kept isolated until SDS on the next day. During R-SDS phase, we conducted the FR task daily.

### Escitalopram administration

Escitalopram (15 mg/kg per day in deionized water) or vehicle was delivered by oral gavage of for 3 weeks after R-SDS procedures [33]. On the days when mice were subjected to the FR task, escitalopram or vehicle administrations were conducted after the mice completed the testing. During the escitalopram treatment phase, these mice were tested on the FR task daily.

### (R,S)-Ketamine administration

Mice received a single acute intraperitoneal injection of vehicle or (R,S)-ketamine. Based on previous studies [34], the dose was 10 mg/kg. Followed by 24 h, we conducted the FR task to evaluate the effect of (R,S)-ketamine. We used only (R,S)-ketamine.

### Social interaction (SI) test

SI tests were performed inside a dark room with a light intensity of 10 lux in a square-shaped box (40 cm × 40 cm) enclosed by walls 27 cm in height. A wire mesh cylindrical cage (8.0 cm diameter × 24 cm high) was centered against one wall of the arena during all SI sessions. Each SI test included two 150 s sessions (separated by an intersession interval of 30 s) without and with the target ICR mouse present in the mesh cage; these sessions were termed “no-target” and “target” sessions, respectively. In the no-target session, a test mouse was placed in the box and allowed to freely explore the environment. The mouse was then removed from the box. In the 30 s break between sessions, the target ICR mouse was introduced into the mesh cage. The design of the cage allowed the animal to fit its snout and paws through the mesh cage but not to escape from the cage. In the target session, the same test mouse was placed beside the wall opposite to the mesh cage. In each session, the time spent in the interaction zone, extending 8 cm around the mesh cage was analyzed. The SI ratio was computed as the ratio of time spent in the interaction zone in the presence of the target to the time spent there in the absence of the target.

### Immunohistochemistry

Following completion of each experiment, mice were deeply anesthetized with ketamine (100 mg/kg) and xylazine (10 mg/kg) and perfused with 4% paraformaldehyde phosphate-buffer solution. Brains were removed from the skull and postfixed in the same fixative overnight. Subsequently, brains were cryoprotected in 20% sucrose overnight, frozen, and cut at 25 μm thickness on a cryostat. Sections were mounted on silane-coated glass slides (Matsunami Glass). Sections were incubated with the primary antibodies overnight at room temperature. The following antibodies were used: anti-green fluorescent protein (GFP) (1:200, goat polyclonal, Rockland Immunochemicals Inc.). For fluorescence microscopy, sections were treated with species-specific secondary antibodies conjugated to Alexa Fluor 488 (1:1,000, Invitrogen) for 2 h at room temperature. Fluorescent images were obtained with an all-in-one microscope (BZ-X710, Keyence).

### Data analysis

All animals and samples were randomly assigned to the experimental groups. Data collection and analysis were not performed blind to the conditions of the experiments. When optic fiber position, electrode position, or microinjection cannula position was not targeted correctly, we excluded those mice. Fiber photometry data were analyzed using custom-written programs in MATLAB. YC ratio (a ratio of yellow to cyan fluorescence intensity; R) in one session was detrended using cubic spline, and normalized within each trial by calculating the *Z*-score as (*R* − *R*_mean_)/*R*_SD_, where *R*_mean_ and *R*_SD_ were the mean and standard deviation of the YC ratio for 5 s just prior to each TS.

### Statistics

Photometry recordings and behavioral experiments were statistically analyzed using MATLAB and SPSS version 24 (IBM, USA). Normality and equal variances were formally tested. Two-sample comparisons were performed by two-sided unpaired *t* test or two-way repeated-measures ANOVA followed by *post hoc* Bonferroni correction. If the data were not normally distributed, we performed with the nonparametric two-sided Wilcoxon signed-rank test. Multiple group comparisons were performed by Mann–Whitney *U*-test and the significance was set at a familywise false discovery rate (FDR) adjusted *p* value of 0.05. k-means clustering was done using the MATLAB function kmeans. Data distribution was analyzed by Spearman’s rank correlation test. Sample sizes were not predetermined, but our sample sizes are similar to (*n* animals) or higher than (*n* traces) those generally employed in the field [20, 35]. All tests used are specified in the supplementary table.

## Results

### Chronic social defeat stress impairs goal-directed behavior

**Fig. 1:**
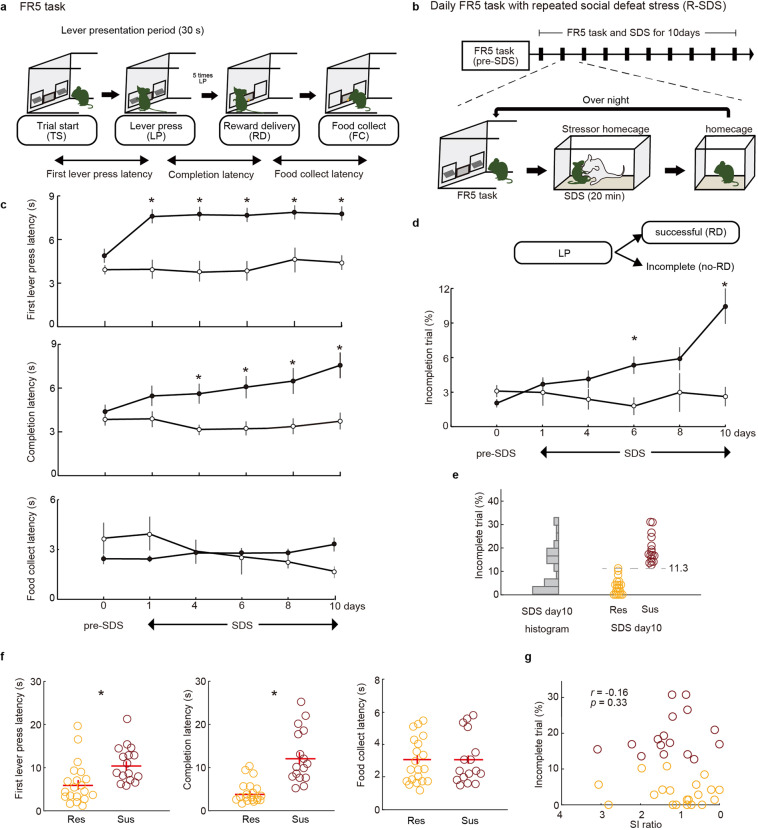
Repeated social defeat stress impairs goal-directed behavior only in stress-susceptible mice. **a** Schematic illustration of the FR-5 schedule. **b** Experimental schedule (upper) and schematic illustration (lower) of operant training during repeated social defeat stress (R-SDS). **c** The effects of repeated social defeat stress (*n* = 36, black circles) on first lever-press latency (top), completion latency of FR-5 (middle), and food collection latency (bottom) in the FR-5 task. Controls (without R-SDS, *n* = 12, white circles) showed stable task performance. Two-way repeated-measures ANOVA confirmed significant group × session interactions on first lever-press latency (*p* = 0.004) and completion latency (*p* = 0.041) but not food collection latency (*p* = 0.737). Bars represents s.e.m. Asterisks (*p < 0.01) represent significant *t*-test with Bonferroni correction (compared with the same time point). **d** Mice receiving R-SDS exhibited a gradual increase in incomplete trials in the FR-5 task (*n* = 36 mice) compared with controls. Two-way repeated-measures ANOVA confirmed a significant group × session interactions on incomplete trials (*p* = 0.031). *p < 0.01, *t*-test with Bonferroni correction. **e** Histogram of incomplete trial% at SDS day 10 indicates a bimodal distribution. Defeated mice were categorized as susceptible (*n* = 16) or resilient (*n* = 20) based on incomplete trials (k-means clustering, *k* = 2). Threshold value dividing susceptible and resilient groups was 11.3%. **f** First lever-press latency and completion latency in susceptible mice were increased (resilient mice, *n* = 20; susceptible mice, *n* = 16 mice), but the food collection latency in FR-5 task was not changed. *p < 0.05 (*t*-test)**. g** The percentage of incomplete trials did not correlate with SI ratio (*n* = 36 mice).

To monitor goal-directed motivated behavior, we used a food-seeking lever-press operant task. In this task, male mice were trained to press a lever five times to obtain one palatable pellet within 30 s (FR-5; Fig. 1a). We previously demonstrated that performance is sensitive to outcome devaluation [20], indicating that behavior is goal-directed in this task. Furthermore, behavioral performance (i.e., success rate) tracked the motivational state and was stable across time in individuals (Fig. 1c, d) [20]. To investigate the effects of stress in this task, we employed a R-SDS model, which is a validated pre-clinical model for major depressive disorder [10, 36]. Each day for 10 consecutive days, we evaluated performance on the FR-5 task and then, immediately after the task, exposed each mouse to a larger aggressive mouse for 20 min (Fig. 1b). Latency to the first LP on each trial (the period from TS to 1st LP) was used as a measure of action initiation. Latency was prolonged beginning 1 day after the start of SDS and remained so for all 10 days of R-SDS (Fig. 1c, top). The number of incomplete trials in which mice did not complete 5 LPs within 30 s gradually increased during R-SDS (Fig. 1d). Completion latency (LP to RW delivery [RD]), a measure of action sustainment, was also increased during R-SDS (Fig. 1c, middle). The total trial number and the success trial ratio were decreased, and the omission trial ratio was increased during R-SDS (supplementary Fig. S1). These data demonstrated that chronic stress exposure reduced motivated behavior. Food collection latency was not altered during R-SDS (Fig. 1c, bottom), suggesting that the preference for palatable pellets after stress exposure was comparable to that before stress exposure. The percentage of incomplete trials in the FR-5 task did not correlate with locomotor activity after R-SDS exposure (supplementary Fig. S2), suggesting that the reduction of FR-5 task performance was not attributable to general changes in activity.

Previous reports demonstrate that mice exposed to R-SDS can be divided into susceptible and resilient populations [10]. Indeed, approximately half of R-SDS-exposed mice exhibited a decreased SI ratio as compared to mice that were not stressed (supplementary Fig. S3). We examined whether resilient and susceptible populations were also identifiable based on goal-directed motivated behavior. We classified defeated mice into two categories, susceptible and resilient, based on their percentage of incomplete trials using a k-means clustering algorithm with *k* = 2 (Fig. 1e). The susceptible population exhibited increased latencies to the first LP and to task completion, whereas stress did not affect these measures in the resilient population (Fig. 1f). We next examined whether stress effects on social behavior predicted the effects of stress on motivated behavior. We found no significant correlations between SI ratio and behavioral parameters of the FR-5 task (Fig. 1g, Supplementary Fig. S4). In summary, results indicate that R-SDS decreases motivation on average, but the population includes susceptible and resilient subgroups. Furthermore, susceptibility to the stress-induced motivational impairments was unrelated to susceptibility to stress-induced social avoidance, suggesting that these two consequences of stress have distinct mechanisms.

### Stress-susceptible mice exhibit increased ventral CA1 activity during goal-directed behavior

**Fig. 2:**
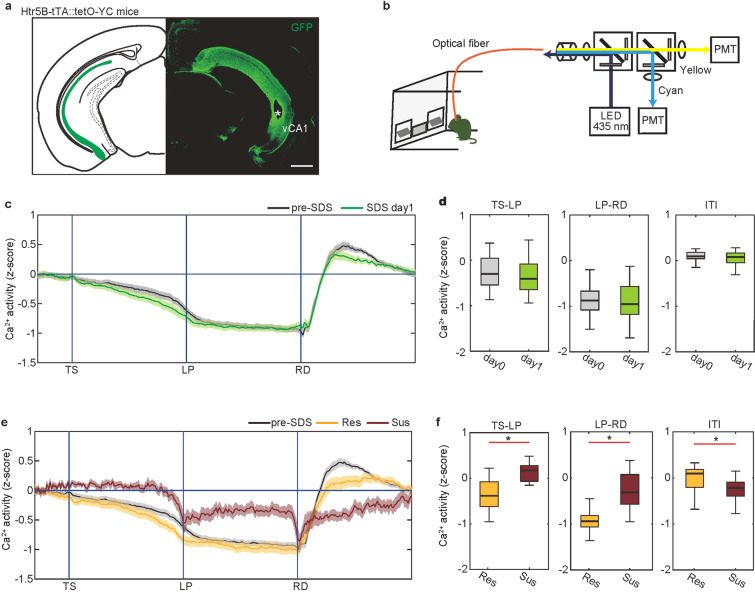
Insufficient vCA1 suppression in mice susceptible to stress-induced motivational impairments. **a** YCnano50 expression (green) in the vCA1 of Htr5B-YC mice. The white asterisk denotes optical fiber placement. Scale bar, 1 mm. **b** Ratiometric fiberphotometry system. YCnano50 was excited with 435 nm. Cyan and yellow fluorescent signals were detected by respective PMTs. **c** Trace of averaged Ca^2+^ signals in the vCA1 of Htr5B-YC mice (*n* = 24 mice) in which the duration between trigger points was normalized. vCA1 activity during the FR-5 task 1 day after the first SDS exposure (day 1, green) did not differ from activity before stress exposure (day 0, black). The shaded areas represent s.e.m. **d** Boxplot representing the averaged Ca^2+^ signal during the TS-LP, LP-RD, and ITI periods of (**c**), respectively (*n* = 24 mice). vCA1 activity in SDS day 1 was comparable to that in pre-SDS. In box plots, the central mark indicates the median and the bottom and top edges of the box indicate the 25th and 75th percentiles, respectively. Whiskers denote the range. **e** Trace of averaged Ca^2+^ signals in vCA1 of resilient (yellow, *n* = 11 mice) or susceptible mice in SDS day 10 (brown, *n* = 9 mice) in which the duration between trigger points was normalized. The shaded areas represent s.e.m. The black trace shows pre-SDS in (**c**). **f** Boxplot representing the averaged Ca^2+^ signal during the TS-LP, LP-RD, and ITI period of (**e**), respectively (resilient, *n* = 11 mice; susceptible, *n* = 9 mice). vCA1 activity in susceptible mice was significantly elevated compared with resilient mice. *p < 0.05 (*t*-test).

We recently demonstrated that the ventral hippocampal CA1 (vCA1) controls action sustainment during goal-directed behavior [20]. Accordingly, we examined the correspondence between vCA1 activity and behavioral change during R-SDS. To monitor vCA1 activity in the task, we used transgenic mice harboring a Ca^2+^ indicator, Yellow Cameleon nano50 (YC), in hippocampal CA1 pyramidal neurons (Htr5B-YC mice, Fig. 2a, Supplementary Fig. S5) and applied fiber photometry [20, 31] to record compound Ca^2+^ signals from vCA1 in freely moving mice (Fig. 2b). Before stress exposure (no-SDS), the temporal pattern of vCA1 activity was as follows: vCA1 activity gradually decreased during the preparatory period (TS-LP), was sustained at the lowered level during lever pressing (LP-RD), rapidly increased after RD, and then overshot and returned to the baseline (Fig. 2c, e, black trace).

All Htr5B-YC mice were exposed R-SDS. We monitored vCA1 activity during the FR-5 task following the first SDS exposure (1 day after) (Fig. 2c, d, green trace) and following the 10th SDS exposure (10 day after) (Fig. 2e, f). Although the latency to the first LP was delayed following a single stress exposure (Fig. 1c), we did not find any effect of stress on vCA1 activity (Fig. 2c, d) at this time point, consistent with prior evidence that vCA1 activity does not modulate action initiation [20, 35]. On the other hand, after 10 days of repeated stress exposure, we found significant changes of vCA1 activity only in mice classified as stress-susceptible based on incomplete trial percentage (Fig. 2e, f, brown trace). Among these mice, vCA1 compound Ca^2+^ levels did not decline during the TS-LP period, the reduction of Ca^2+^ levels during the LP-RD period was less pronounced than in no-SDS controls, and the rise of Ca^2+^ levels after RD was attenuated (Fig. 2e, f). Of note, an insufficient reduction of vCA1 activity during lever pressing was previously associated with prolonged latency to task completion [20]. The vCA1 activity of resilient mice was comparable to that of no-SDS controls. These results indicate that task-related vCA1 hyperactivity coincides with stress-induced impairment of successful goal-directed behavior.

Next, we asked whether stress-induced social withdrawal is related to the motivational impairments we observed. We classified the same R-SDS-exposed mice as resilient or susceptible based on SI ratio and then compared motivated behavior and vCA1 compound Ca^2+^ levels between these two groups. Surprisingly, goal-directed behavior did not differ between mice that were resilient to social withdrawal and those that were susceptible to social withdrawal (Supplementary Fig. S6). In addition, there were no differences between these two groups in vCA1 activity during the LP-RD period of the operant task (Supplementary Fig. S6). These data suggest that stress-induced motivational impairments are mechanistically distinct from susceptibility to social withdrawal.

### Antidepressants restore both vCA1 activity dynamics and motivation after stress

**Fig. 3:**
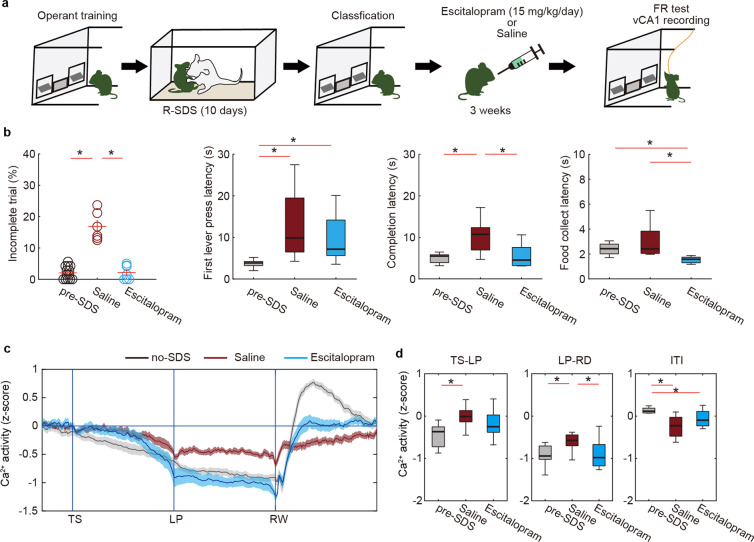
Chronic SSRI treatment normalized both motivation and ventral hippocampal activity after stress. **a** Design of the experiment. **b** The effects of chronic escitalopram treatment on behavior (pre-SDS, *n* = 13 mice; saline, *n* = 6 mice, escitalopram, *n* = 7 mice) in the FR-5 task. *p < 0.05 (Mann–Whitney *U*-test with FDR correction). **c**, **d** The effect of chronic escitalopram treatment on Ca^2+^ activity in vCA1 during the FR-5 task (pre-SDS, *n* = 13 mice; saline, *n* = 6 mice, escitalopram, *n* = 7 mice). Trace of averaged Ca^2+^ signals in which the duration between trigger points was normalized (**c**). Boxplot representing the averaged Ca^2+^ signal during TS-LP, LP-RD, and ITI periods of (**c**), respectively (**d**). *p < 0.05 (Mann–Whitney *U*-test with FDR correction). Bars represent the mean and lines represent the s.e.m. The shaded areas represent s.e.m. In box plots, the central mark indicates the median and the bottom and top edges of the box indicate the 25th and 75th percentiles, respectively. Whiskers denote the range.

We next addressed whether R-SDS-induced motivation deficit could be treated using antidepressant drugs. After chronic stress exposure and classification of mice based on the percentage of incomplete trials, we administered escitalopram (selective serotonin reuptake inhibitor [SSRI]) to susceptible mice for 3 weeks (Fig. 3a). During SSRI treatment, we monitored behavioral performance on the FR-5 task. We found that 3 weeks of SSRI treatment restored both the trial completion percentage and the completion latency but not the latency to the first LP in the FR-5 task (Fig. 3b), suggesting that SSRI targeted an action sustainment component of the behavior. We therefore monitored the corresponding vCA1 patterns during the behavioral task and found that chronic SSRI treatment restored the suppression of vCA1 activity during lever pressing (LP-RD period) (Fig. 3c, d), suggesting that the therapeutic effect of SSRI was mediated by the normalization of vCA1 activity during sustained goal-directed action.

**Fig. 4:**
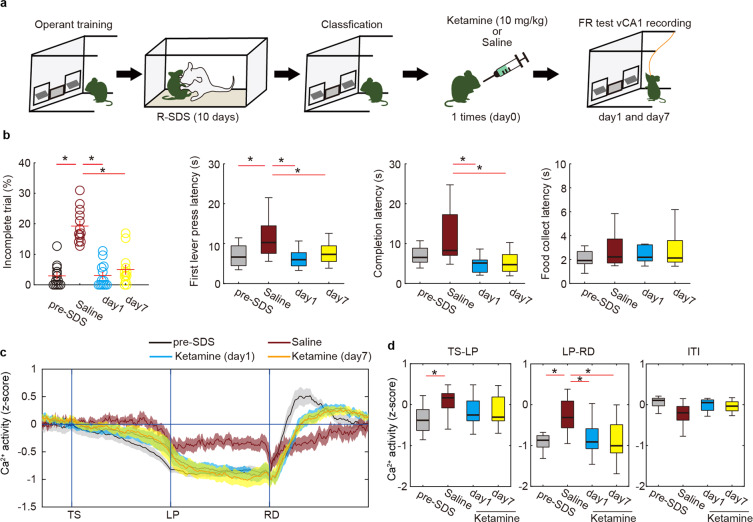
Rapid and long-term effects of a single ketamine injection on motivation and ventral hippocampal activity after stress. **a** Schematic illustration of ketamine treatment. **b** The effects of ketamine treatment on behavior (base, *n* = 13 mice; saline, *n* = 13 mice; ketamine (day 1), *n* = 13 mice; ketamine (day 7), *n* = 13 mice) in the FR-5 task. *p < 0.05 (Mann–Whitney *U*-test with FDR correction). **c**, **d** The effect of ketamine treatment on Ca^2+^ activity in vCA1 during the FR-5 task (base, *n* = 13 mice; saline, *n* = 13 mice; ketamine (day 1), *n* = 13 mice; ketamine (day 7), *n* = 13 mice). Trace of averaged Ca^2+^ signals in which the duration between trigger points was normalized (**g**). Boxplot representing the averaged Ca^2+^ signal during TS-LP, LP-RD, and ITI periods of (**c**), respectively (**h**). *p < 0.05 (Mann–Whitney *U*-test with FDR correction). Bars represent the mean and lines represent the s.e.m. The shaded areas represent s.e.m. In box plots, the central mark indicates the median and the bottom and top edges of the box indicate the 25th and 75th percentiles, respectively. Whiskers denote the range.

Recent clinical studies confirm that ketamine is effective as a rapidly acting antidepressant [37–39]. In rodents, ketamine is effective against R-SDS-induced depression-like behavior [40]. We sought to address whether ketamine could ameliorate stress-induced motivation impairments and normalize vCA1 activity, similar to chronic SSRI treatment. We administered a single ketamine injection with a subanesthetic dose to susceptible mice and monitored behavioral performance and vCA1 activity during the FR-5 task 1 and 7 days after the treatment (Fig. 4a). At both time points, a single ketamine treatment restored all behavioral parameters that had been altered after R-SDS (Fig. 4b), demonstrating rapid and long-lasting effects of ketamine in our mouse model. Notably, ketamine rescued deficits in both action initiation and sustainment. During action sustainment (LP-RD period), ketamine reversed the stress-induced increase in vCA1 activity at both 1 and 7 days after treatment (Fig. 4c, d), suggesting that the behavioral effect of ketamine was mediated by modulation vCA1 activity dynamics.

To address whether ketamine modulates motivated behavior under unstressed conditions, we trained mice to press lever to earn food on a FR-10 schedule, in which more effort was required and, as a consequence, the incomplete trial percentage was higher than in the FR-5. We administered a single ketamine injection to unstressed mice and monitored behavior and vCA1 activity. There were no significant effects of ketamine on behavior or vCA1 activity under either the FR-10 or FR-5 schedules (Supplementary Fig. S7). These data indicate that the effects of ketamine are unique to stressed animals.

**Fig. 5:**
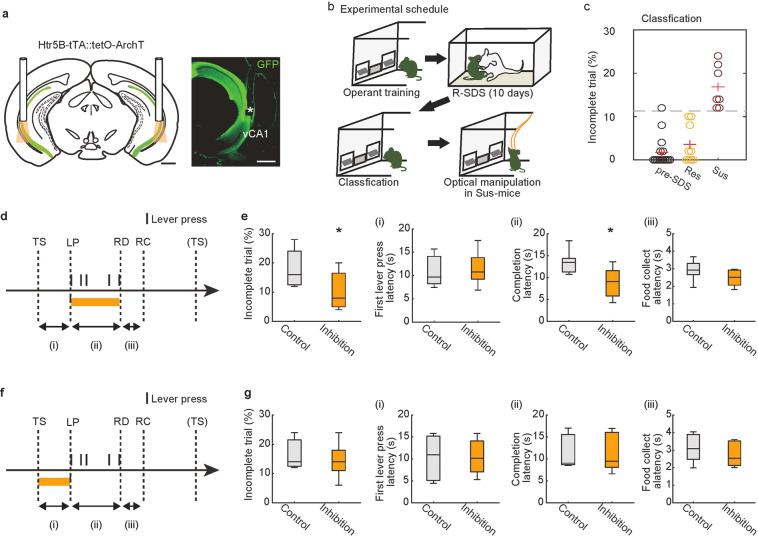
Artificial suppression of vCA1 activity rescues stress-induced motivational impairments. **a** Bilateral optogenetic inhibition of vCA1 was performed in Htr5B-ArchT mice (left). Expression of ArchT-EGFP in vCA1 of Htr5B-ArchT mouse. The white asterisk denotes optical fiber placement. Scale bar, 1 mm. **b** Design of optogenetic experiment. **c** Defeated mice (*n* = 16 mice) were categorized into resilient (*n* = 9) or susceptible (*n* = 7) based on incomplete trials (Threshold is 11.3 %, gray dashed line). **d**, **f** Timing of illumination. Optogenetic inhibition was conducted during every LP-RD (**d**) or TS-LP (**f**) period. **e** The effects of vCA1 inhibition in susceptible mice during the LP-RD period of the FR-5 task (*n* = 7 mice). *p < 0.05, two-sided Wilcoxon signed-rank test. **g** The effects of vCA1 inhibition in susceptible mice during the TS-LP period of the FR-5 (*n* = 7 mice). *p < 0.05, two-sided Wilcoxon signed-rank test. In box plots, the central mark indicates the median and the bottom and top edges of the box indicate the 25th and 75th percentiles, respectively. Whiskers denote the range.

The SSRI and ketamine experiments suggest normalization of vCA1 activity during lever pressing may be a shared therapeutic mechanism for stress-induced motivation impairments. To test this hypothesis, we investigated whether temporally specific augmentation of vCA1 suppression is sufficient to rescue impairments in goal-directed behavior caused by R-SDS. For vCA1 inhibition, we used transgenic mice (Htr5B-ArchT mice), in which only the CA1 pyramidal neurons expressed an inhibitory opsin (ArchT) (Fig. 5a, supplementary Fig. S8). After stress exposure, susceptible mice were identified as above based on the percentage of incomplete trials. We then evaluated the effects of bilateral optogenetic inhibition of vCA1 activity on FR-5 task performance (Fig. 5b, c). Inhibition of vCA1 during the LP-RD period reduced the percentage of incomplete trials and the completion latency in R-SDS-exposed mice (Fig. 5d, e). This manipulation did not affect latency to the first LP or latency to food collection (Fig. 5e), confirming prior evidence for a specific role of vCA1 in action sustainment [20]. Inhibition of vCA1 during the TS-LP period did not affect behavioral parameters (Fig. 5f, g), emphasizing the temporal specificity of vCA1 suppression. vCA1 inhibition did not increase locomotor activity (Supplementary Fig. S9), suggesting that the increase in motivated behavior was not due to hyperlocomotion. Taken together, our results suggest that R-SDS prevents vCA1 suppression during sustained goal-directed behavior, thereby impairing of action sustainment and reducing success of goal-directed behavior. After stress, chronic SSRI treatment or a single ketamine injection restores motivated behavior via normalization of vCA1 activity during action sustainment.

## Discussion

Our results demonstrate that chronic stress exposure impairs goal-directed motivated behavior in mice. During 10 days of R-SDS, mice lever pressing for food RW displayed increases in the number of incomplete trials, the latency to start lever pressing, and the latency to complete a trial. In contrast, the latency to collect the food RWs, once earned, was unaffected by stress, suggesting that motivational deficits do not reflect blunted hedonic RW. Stress-induced motivational impairments were associated with hyperactivity of the vHP during periods when effortful responding was required. The behavioral deficits and hippocampal hyperactivity were corrected by each of two antidepressant medications, the SSRI escitalopram and ketamine.

The hippocampus is believed to play important roles in the stress response and the pathology of depression [18, 41]. Hyperactivity of vHP is associated with negative-valence states such as fear and anxiety, and hyperactivity of vHP impairs goal-directed behavior [20]. Stress-exposed rodents show increased glutamate release [42], increased glutamate receptor expression [43], and facilitated induction of *c-fos* and *Arc* mRNA expression in the hippocampus [44]. Artificial enhancement of glutamatergic transmission from vHP to nucleus accumbens induced susceptible phenotype after chronic defeat stress [45]. The ventral extent of the hippocampus is particularly sensitive to the effects of stress [15–17]. These observations may account for the hyperactivity of vHP after stress exposure and the insufficient decline of vHP activity during goal-directed behaviors.

Our data demonstrate that vHP dysregulation plays a direct causal role in stress-induced motivational deficits and that the vHP activity is a shared target of antidepressants of different classes. The suppressive effects of these drugs on vHP activity may be mediated by direct or indirect serotonin-mediated inhibition [20, 46], ketamine-mediated NMDA receptor blockade [37, 40], or ketamine-mediated serotonin uptake inhibition [47]; however, more detailed investigations will be necessary to explain the temporal specificity of the suppressive effects.

A recent study demonstrated that RW behavior is modulated by the strength of vHP to nucleus accumbens synapses [48]. In that study, chronic stress reduced the strength of vHP-accumbal dopamine receptor type1-expressing medium spiny neuron (D1-MSN) synapses but not D2-MSN synapses, and SSRI treatment rescued it [48]. Our data demonstrate vHP hyperactivity under chronic stress exposure, but why bulk vHP hyperactivity would selectively weaken vHP-accumbal D1-MSN synaptic strength is unclear. One possibility is that the bulk Ca^2+^ activity that we monitored is insensitive to differences in activity among specific projecting populations (e.g., D1-targeting versus D2-targeting vCA1 cells). It will be valuable to monitor projection-specific neural activity during motivated behavior to determine whether changes in activity dynamics are confined to particular projection pathways. Although the two studies highlight different circuit mechanisms, the LeGate et al. [48] study and our own converge on vCA1 as an important target for understanding stress and antidepressant effects on motivated behavior.

Our results suggest that acute and chronic stress have distinct motivational effects. On day 1 after stress exposure, mice exhibited prolonged latency to the first LP, while measures of action sustainment remained were unaffected. The latency to the first LP reflects the action initiation component in effortful goal-directed behaviors [31] and is modulated by an insular cortex-ventral striatum pathway [35]. Interestingly, stress-induced increases in first LP latency were responsive to ketamine treatment (Fig. 5b) but not SSRI treatment (Fig. 4b). These pharmacological responses suggest that an acute-onset and sustained action initiation impairment is not mediated by the dysregulation of the ventral hippocampal activity. Insular cortex is known to mediate the retrieval of the outcome value [49] and to guide behaviors on the basis of anticipation of food [50]. These processes may preferentially be affected by acute stress and impair initiation of goal-directed behaviors. Further studies are needed to dissociate circuitries targeted by acute and chronic stress.

Because multiple systems—endocrine, immune, autonomic, emotional, and cognitive—are involved in the stress response, it is plausible that stress resilience and susceptibility are multi-dimensional processes with the potential for orthogonal outcomes at each of several potentially stress-responsive loci. Social withdrawal is one well-known sequela of R-SDS, and numerous studies have validated the susceptible vs resilient dichotomy with respect to this outcome in the R-SDS model [51–56]. Our data suggest that impaired goal-directed motivation constitutes an independent dimension of the stress response, and susceptibility and resilience at the level of motivation is orthogonal to an individual’s classification with respect to social behavior. We hypothesize that stress-induced social and motivational impairments have distinct circuit mechanisms, with a vHP-mediated emotional and cognitive system shaping responsiveness in the motivational dimension (our study) and a PFC/amygdala-mediated system controlling that in the social dimension [32, 57] in the R-SDS model. Our findings favor the view that effective treatments for stress-related psychopathology will require treatment approaches tailored to an individual’s particular pattern of susceptible and resilient behavioral domains and their associated neural circuits.

In addition to establishing a link between stress and goal-directed behavior, the paradigm established here may have general utility as a model for investigating treatments and mechanisms of motivational disorders. According to the framework put forth by Nestler and Hyman [58], animal models of neuropsychiatric conditions should be evaluated by reference to three forms of validity. Face validity of the current model derives from the fact that the operant lever pressing-deficits task is readily translatable to humans, and, in fact, similar lever pressing impairments have been observed in patients with major depression [8]. Pharmacological validity refers to a model’s ability to accurately discriminate effective versus ineffective therapeutics. Although we have not established that the current model can identify novel antidepressant therapeutics, which is the strongest test of pharmacological validity, we are encouraged by the fact that the current model is sensitive to two antidepressants of different classes. Construct validity refers to homology between the environmental or biological conditions that produce the disease. In humans, stressful life events have a well-established causal effect on depression onset [23]. As for biological constructs, it is unknown whether task-related vHP hyperactivity of the kind reported here is present in human patients, but this phenotype is amenable to analysis in humans, provided that imaging modalities such as fMRI or PET can provide adequate temporal precision.

One caveat of this experiment is that calorie restriction by itself can produce anti-depressive effects [59]. Since food restriction is required for good performance in our operant task, it would be difficult to investigate whether food restriction moderated the effects of stress in this task. However, we can conclude that food restriction was not sufficient to prevent effects of R-SDS on motivation, nor was it sufficient to prevent the effects of our antidepressant manipulations. A related possibility is that changes in operant performance in our task reflect changes in hunger/satiety. This is unlikely, however, because food collection latency was not affected by our manipulations, indicating that mice still desired the food when it was available.

In conclusion, our data identify vCA1 activity dynamics as a putative biomarker for stress-induced motivational deficits. vCA1 activity differentiates susceptible versus resilient individuals and is responsive to antidepressant treatment. Furthermore, optogenetic stimulation demonstrates a causal effect of vHP activity on goal-directed behavior. The fact that vCA1 activity responded to antidepressant drugs with distinct primary pharmacological targets suggests that vCA1 is a higher-order therapeutic target that might be modulated via various signaling pathways. The results encourage the use of vCA1 dynamics as a circuit-level biomarker that might be used to accelerate discovery of drugs targeting motivational impairments.

## Funding and disclosure

This work was supported by Grant for Research Fellow of the Japan Society for the Promotion of Science (20J00643) to KY, Grant-in-Aid for Scientific Research on Innovative Area “Willdynamics” (19H05027) from the MEXT to KFT, Grant-in-Aid for Brain Mapping by Integrated Neurotechnologies for Disease Studies (Brain/MINDS) (JP20dm0207069) from AMED to KFT, and Grant-in-Aid for Program for the Advancement of Next Generation Research Projects from Keio University to KFT. The authors declare no competing interests. We dedicate this paper to the memory of Dr. Keitaro Yoshida, an immensely talented graduate student who passed away while he was spearheading this project.

## Supplementary information

supplementary figures and tables
