# Supplementary material for: Chronic social defeat stress impairs goal-directed behavior through dysregulation of ventral hippocampal activity in male mice
Source: Neuropsychopharmacology. 2021 Mar 10;46(9):1606–16. doi: 10.1038/s41386-021-00990-y (PMC8280175; doi:10.1038/s41386-021-00990-y)
Supplement: Supplementary file 1 — supplementary figures and tables [file 41386_2021_990_MOESM1_ESM.docx]

# Title:

**Chronic social defeat stress impairs goal-directed behavior through dysregulation of ventral hippocampal activity in male mice**

**Authors:**

Keitaro Yoshida^1^, Michael R. Drew^2^, Anna Kono^1^, Masaru Mimura^1^, Norio Takata^1^, Kenji F. Tanaka^1^

**Affiliations:**

^1^Department of Neuropsychiatry, Keio University School of Medicine, Tokyo, Japan,

^2^Center for Learning and Memory, Department of Neuroscience, The University of Texas at Austin, Austin, Texas, USA,

**Corresponding author**:

Kenji F. Tanaka, M. D., Ph. D.

Department of Neuropsychiatry, Keio University School of Medicine, Tokyo 160-8582, Japan.

TEL: +81-3-5363-3934

e-mail address: [kftanaka@keio.jp](mailto:kftanaka@keio.jp)

**
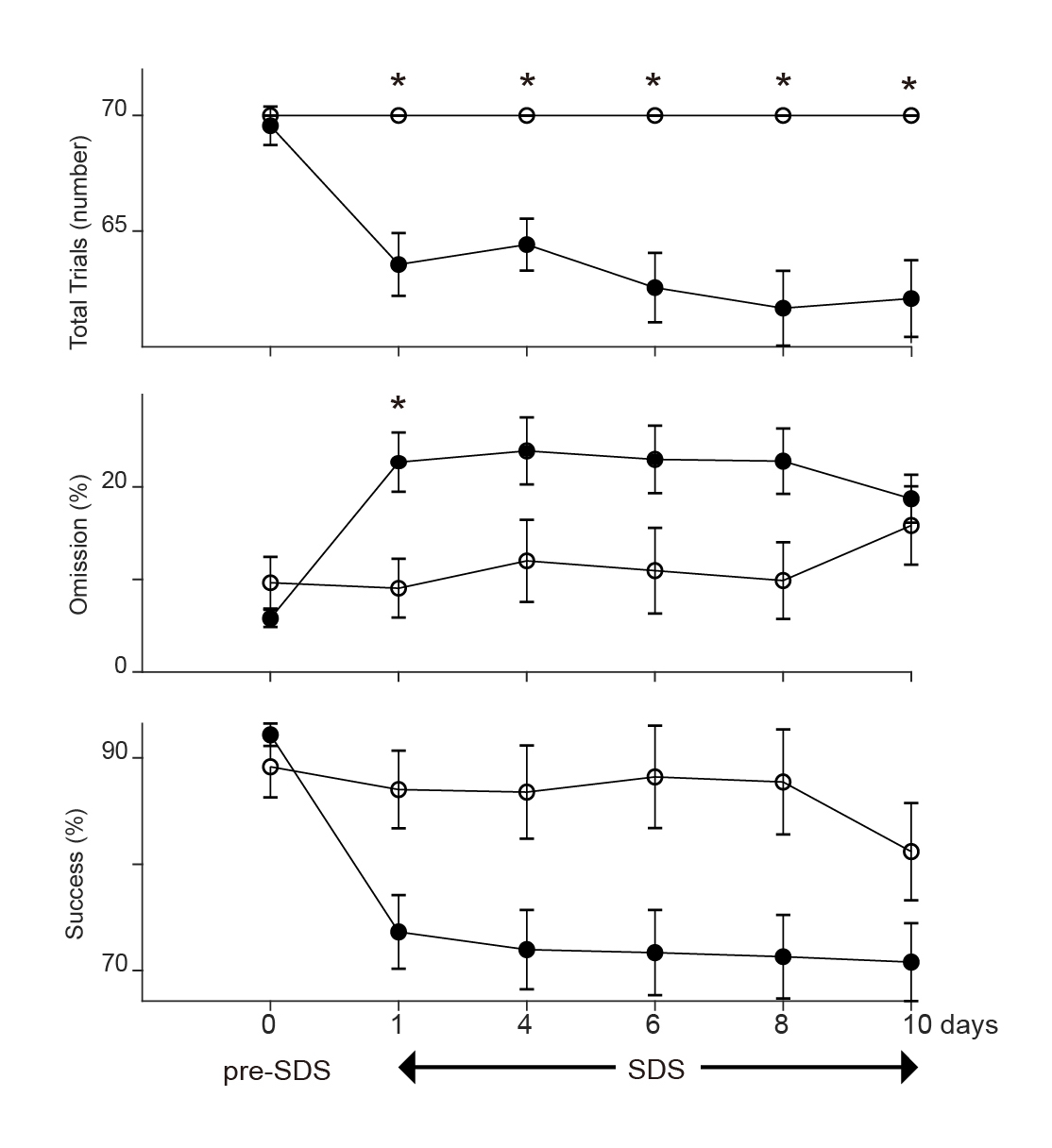
**

**Fig. S1**

**The effect of R-SDS on operant task performance.**

The temporal changes of total trial number, omission trial ratio, and success trial ratio in defeated mice (n=36, black circles) and control mice (n=12, white circles). Two way repeated-measures ANOVA confirmed significant group x session interaction on total trial number (p=0.01), omission trial ratio (p=0.033), and success trial ratio (p=0.013). Bars represent s.e.m. Asterisks (*p<0.01) represent significant t-test with Bonferroni correction.


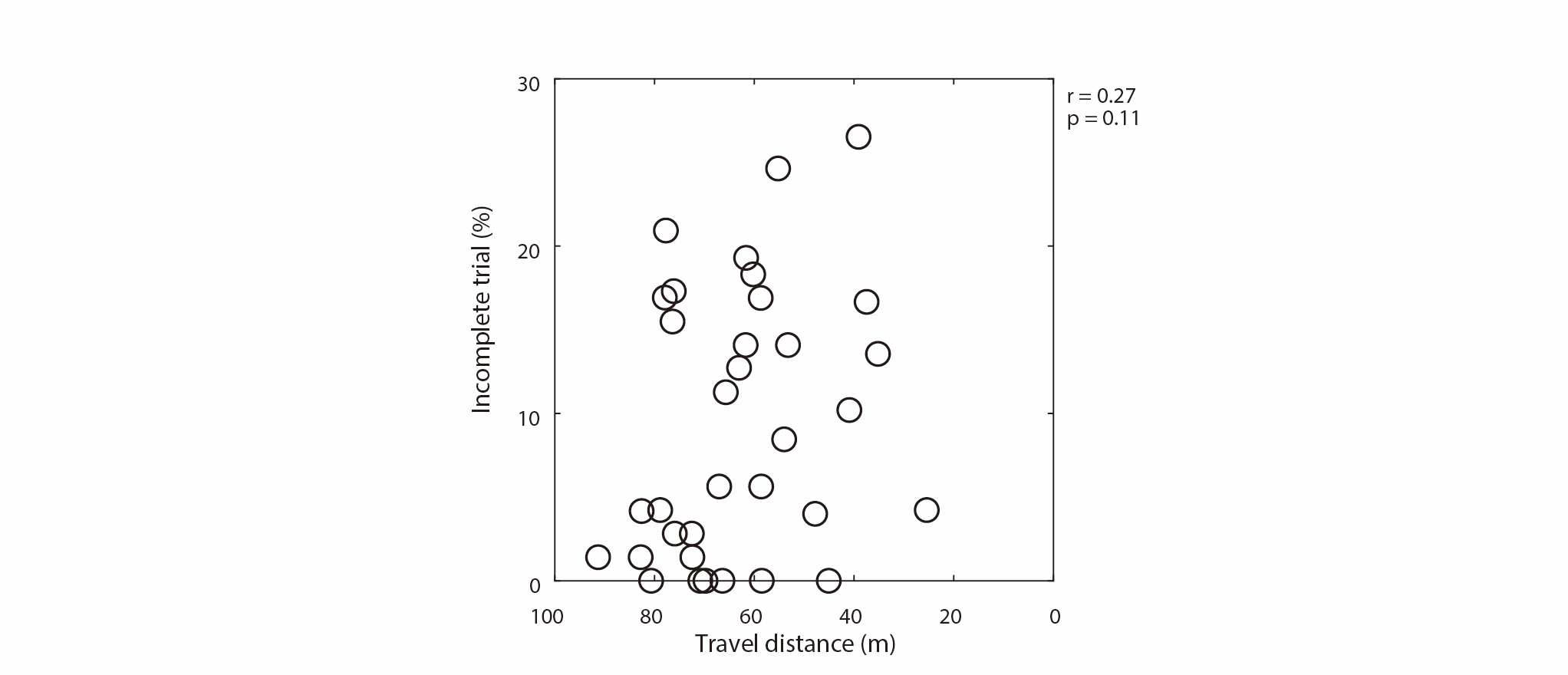


## Fig. S2

## No significant correlation was found between the failure trial ratio and the basal locomotion activity.

To examine the basal locomotion activity, we conducted the open field test for twenty minutes after R-SDS exposure.


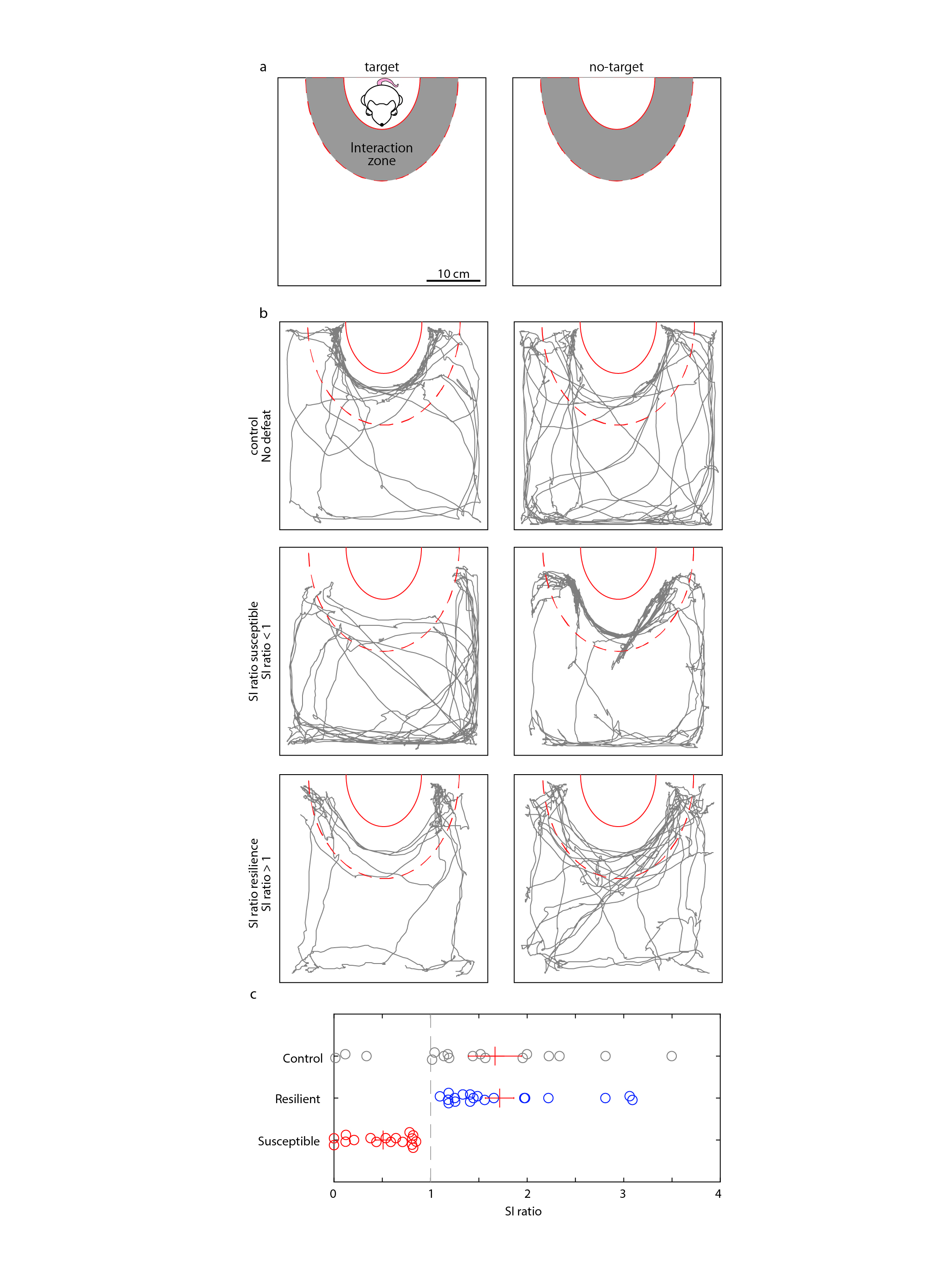


## Fig. S3

## Repeated social defeat results in social withdrawal.

**a**, Schematic illustration of the social interaction (SI) test showing the interaction zone with or without target mice. **b,** Representative position tracking of control (no defeated), susceptible (SI ratio < 1), and resilient (1 < SI ratio) mice. **c,** SI ratio was computed as the ratio of occupancy time in the interaction zone in the target session to that in the no target session (control mice, n = 18; resilient mice, n=19 mice; susceptible mice, n=17 mice). Each circle represents an animal and red crossline represents mean ± s.e.m.


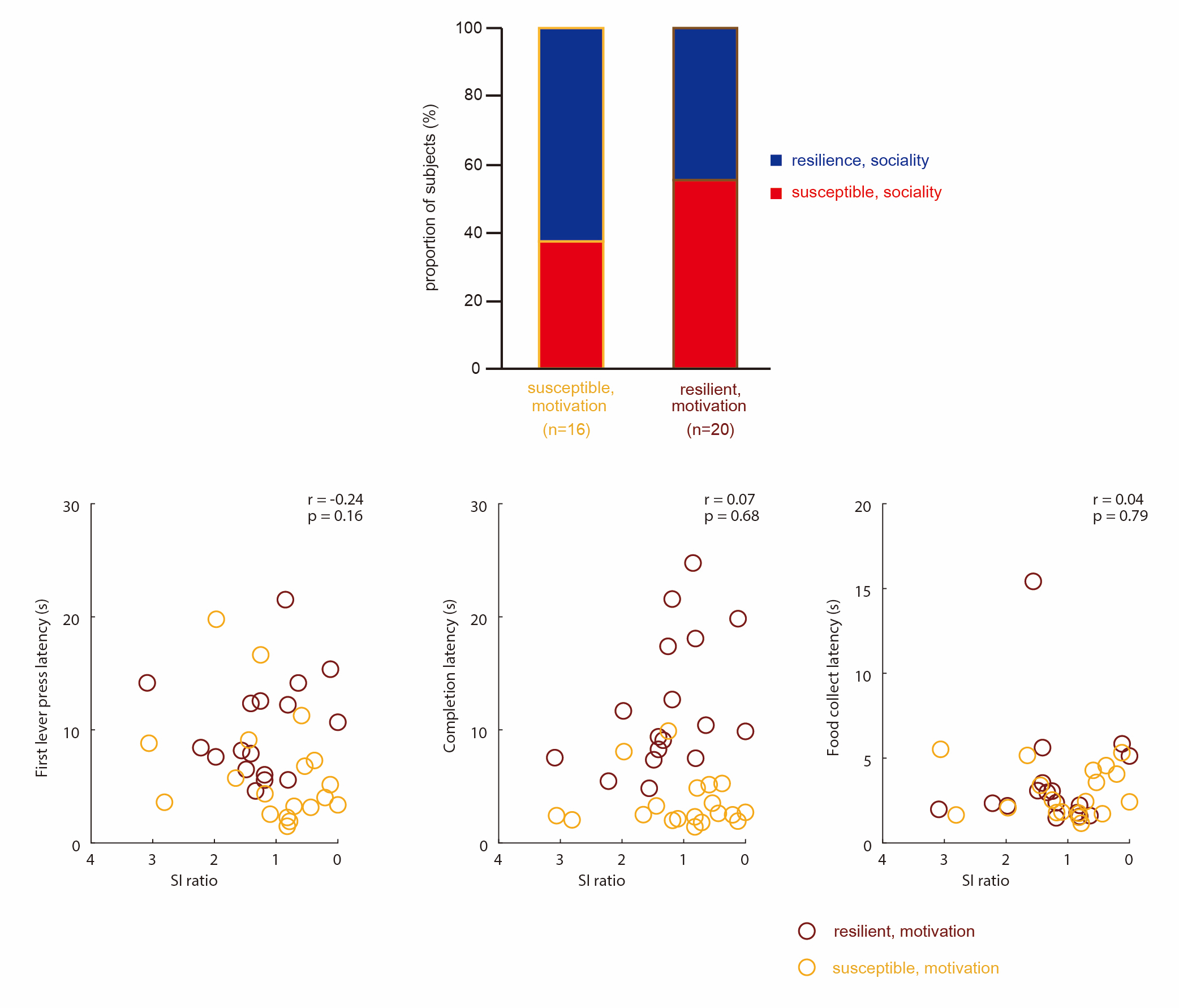


## Fig. S4

## No significant correlations were found between SI ratio in social interaction test and behavioral parameters in FR-5 task.

Upper) Proportion of mice classified as susceptible or resilient based on SI ratio as a function of their classification based on motivation. Among mice classified as resilient to stress-induced motivation impairments, 55% were classified as susceptible to stress-induced social impairments.

Lower) First lever press latency, completion latency of FR-5, and food collect latency of FR-5 task did not correlate with SI ratio of social interaction test (n = 36 mice). Brown and yellow circles represent susceptible (higher failure ratio) and resilient mice (lower failure ratio), respectively.


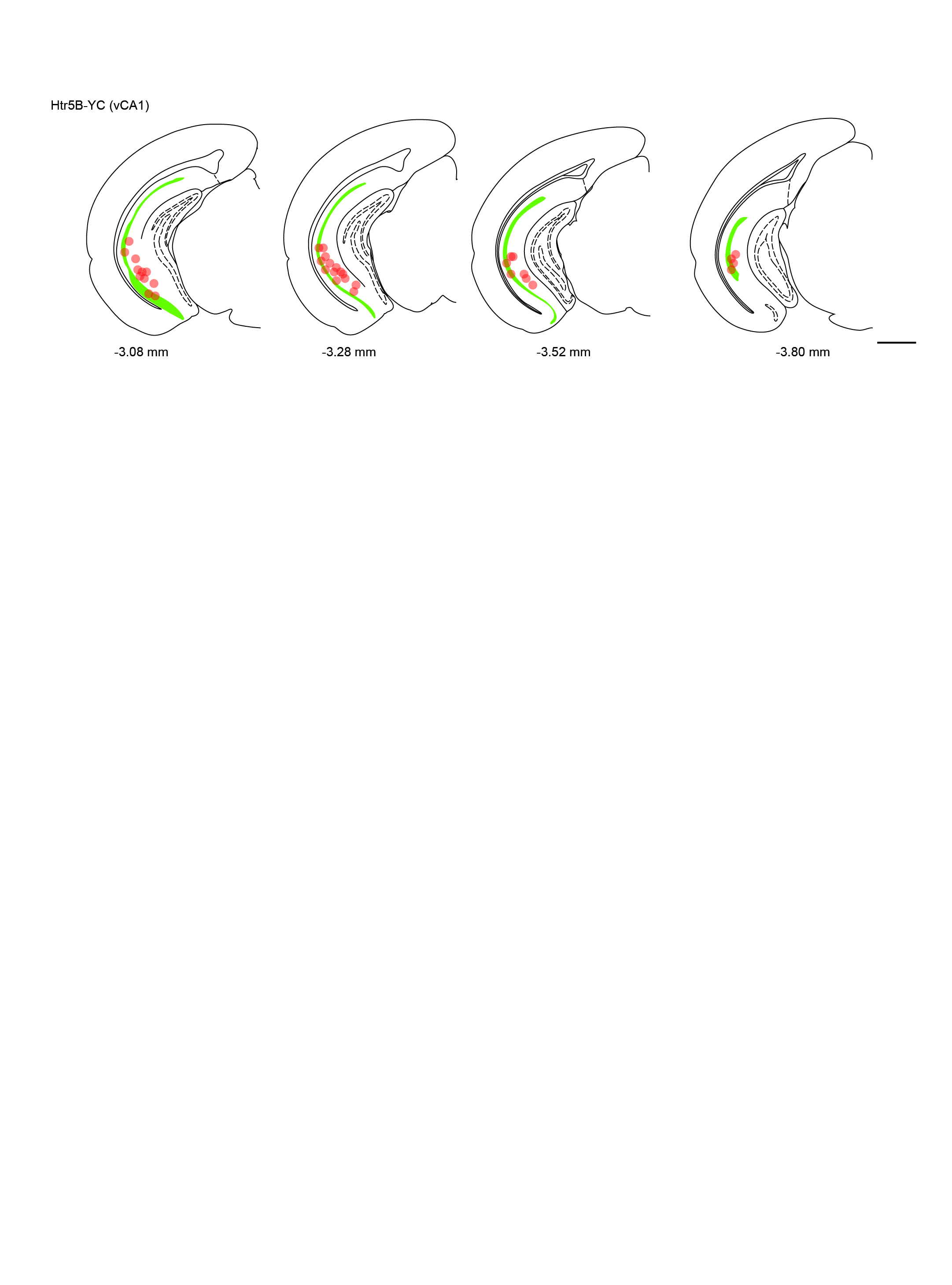


## Fig. S5

## Optical fiber placements for fiber photometry.

Histological reconstruction of optical fiber tip placement of Htr5B-YC mice. Red dots indicate fiber tips. Scale bar, 1 mm.


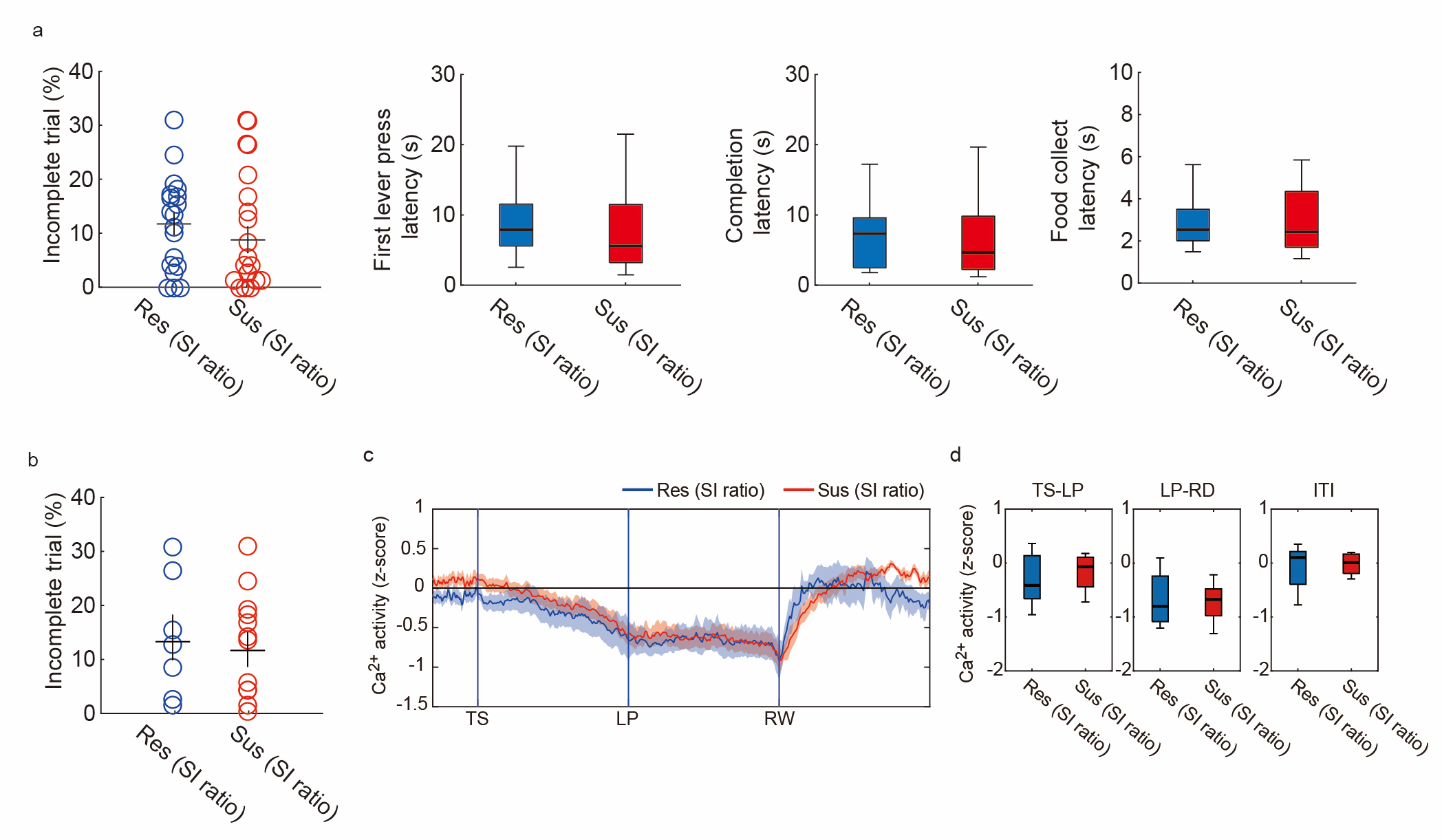


**Fig S6**

**Operant performance and vCA1 Ca^2+^ activity in mice classified as resilient or susceptible based on SI ratio.**

**a**, No difference in behaviors in the FR-5 task (resilient (SI ratio), n=19, susceptible (SI ratio), n=17, related to Fig 1). b, No difference in incomplete trial % in the FR-5 task (resilient (SI ratio), n=7, susceptible (SI ratio), n=12, related to Fig 2). c,d, No difference in Ca^2+^ activity of vCA1in FR-5 task (resilient (SI ratio), n=7, susceptible (SI ratio), n=12). **c,** Trace of averaged Ca^2+^ signals in which the duration between trigger points was normalized. **d**. Boxplot representing the averaged Ca^2+^ signal during the TS-LP, LP-RD, and ITI period, respectively. vCA1 activity was comparable in mice that exhibited SI impairments (susceptible) and those that did not (resilient). Bars represent the mean and lines represent the s.e.m. The shaded areas represent s.e.m. In box plots, the central mark indicates the median and the bottom and top edges of the box indicate the 25th and 75th percentiles, respectively. Whiskers denote the range.


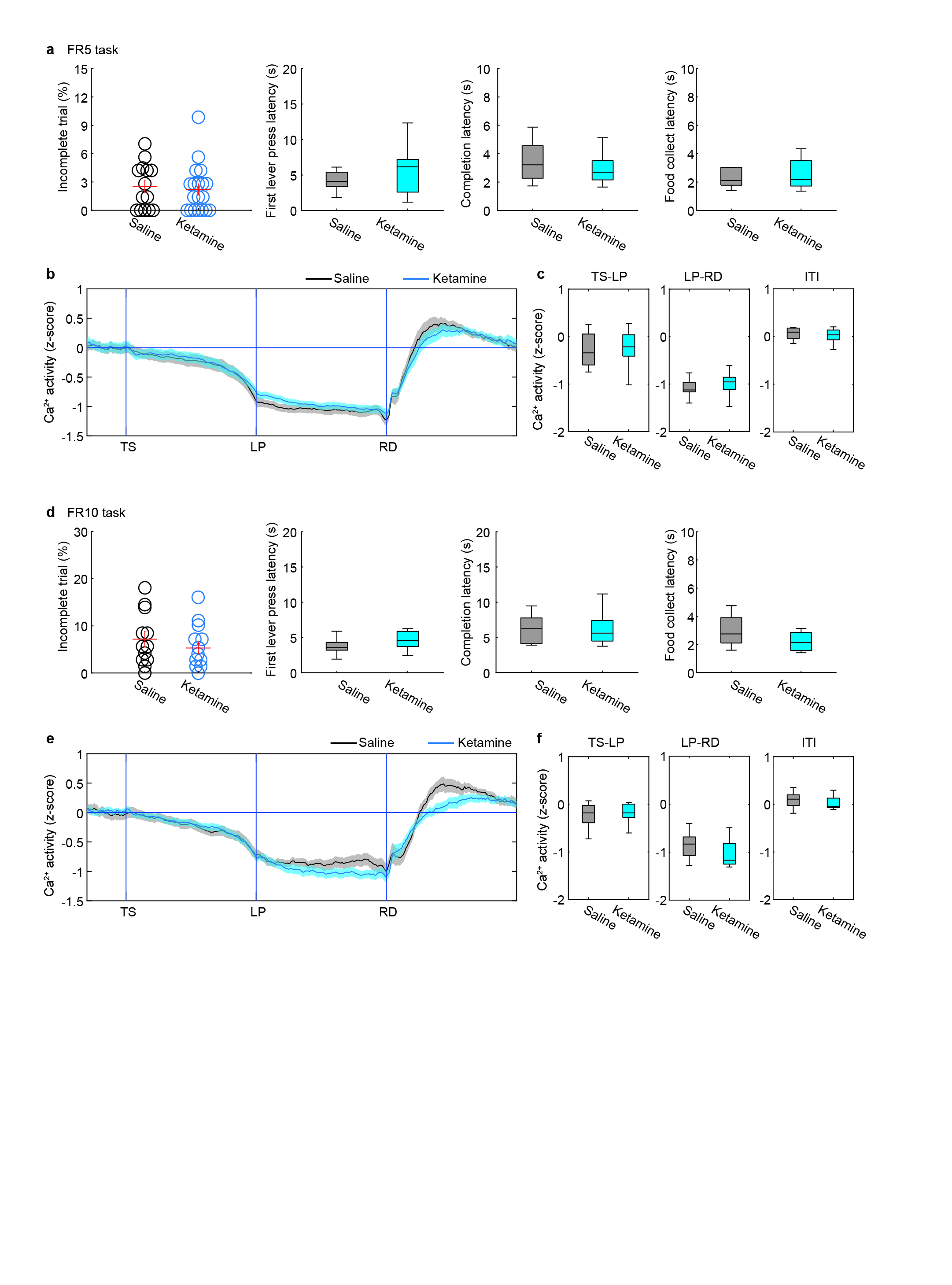


**Fig S7**

## No effects of ketamine on operant performance and vCA1 Ca^2+^ activity in the absence of social defeat stress.

**a**, No effects of ketamine administration on behavior in the FR-5 task of non-stressed mice (saline control, n = 14 mice; ketamine, n = 14 mice). **b, c,** No effect of ketamine administration on Ca^2+^ activity in vCA1 in the FR-5 task (saline control, n = 14 mice; ketamine, n = 14 mice). Trace of averaged Ca^2+^ signals in which the duration between trigger points was normalized (b). Boxplot representing the averaged Ca^2+^ signal during TS-LP, LP-RD, and ITI period, respectively (c). **d**, No effects of ketamine administration on behavior in the FR-10 task of non-stressed mice (saline control, n = 12 mice; ketamine, n = 12 mice). **e, f,** No effect of ketamine administration on Ca^2+^ activity of vCA1in FR-10 task (saline control; n = 12 mice, ketamine; n = 12 mice). Trace of averaged Ca^2+^ signals in which the duration between trigger points was normalized (e). Boxplot represented the averaged Ca^2+^ signal during TS-LP, LP-RD, and ITI periods, respectively (f).

##
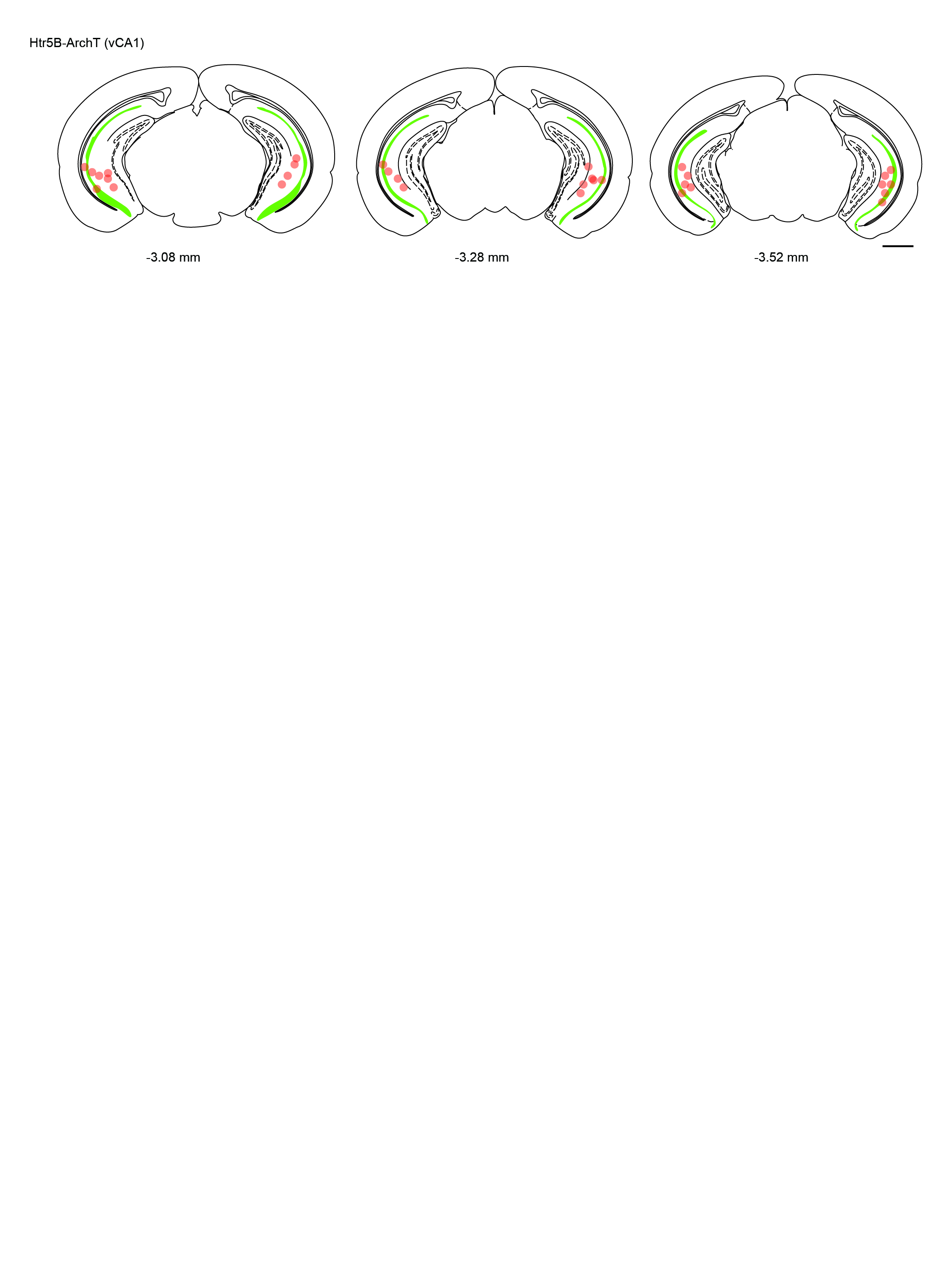


## Fig. S8

## Optical fiber placements for optogenetics.

Histological reconstruction of optical fiber tip placement of Htr5B-ArchT mice. Red dots indicate fiber tips. Scale bar, 1 mm.


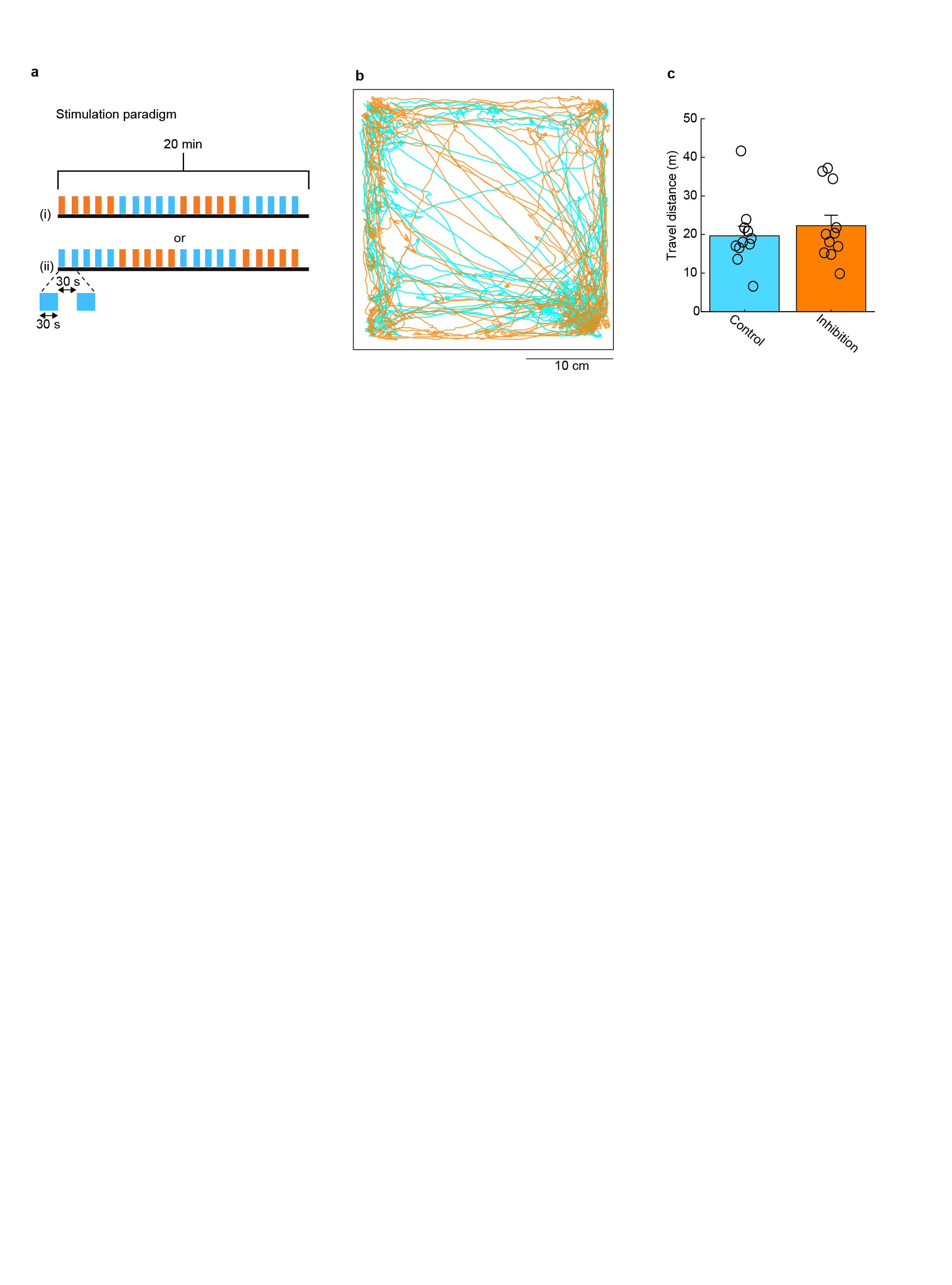


## Fig. S9

## No effect of vCA1 inhibition on locomotor activity.

**a**, Stimulation paradigm. Yellow and blue light illumination trains were delivered (30-s illumination at 30-s interval, 5 pulses) to bilateral vCA1 of Htr5B-ArchT mice. The order of the light train blocks was counterbalanced between mice. **b,** Representative position tracking during the yellow and blue light trains. **c,** Distance traveled during the ten minutes of yellow or blue light illumination (n = 11 mice). Bars represent the mean, lines represent the s.e.m., and each circle represents an animal.

**supplemental table 1: statistics for main figures**

**supplemental table 2: statistics for supplementary figures**
